# Pathological Conditions of the Urine

**Published:** 1904-07-02

**Authors:** 


### Progress in Medicine and Surgery.

### PATHOLOGICAL CONDITIONS OF THE URINE.

(*Concluded from page 227.*)

**Functional Albuminuria** has been much discussed. It has been attributed to incipient renal disease, to vasomotor conditions, and to sexual changes. In some cases it is influenced by diet and may disappear on purely milk food, but in others posture alone makes a change. Samuel West[8] finds that in boys and young adults most cases get well, in some the condition continues without any other sign of bad health, and in a few renal disease developes. Some of the boys are vigorous, with good pulse tension, and others are pale, weakly, and with a low tension pulse; as age advances functional albuminuria increases the risk to life. Over the age of 40 such cases should be refused for insurance, and below that age an additional premium is needed. All cases with thickened arteries should be rejected. Pavey agrees that it does not always end in renal disease. He has watched patients for 15 years. Garrod assents to this, but notes oxaluria as sometimes a cause, and thinks disease of a part of one kidney may also produce it. G. A. Sutherland[9] thinks the postural form occurs in a definite group of cases which show special symptoms of vasomotor disturbance, and not those of renal disease. It is in fact a symptom of neurasthenia and is best treated like other instances of that disorder. In examining for insurance Pye Smith[10] lays down that with young men we must not trust to one test, but try two or more, such as boiling and also the ferrocyanide test. If positive, he would request a second examination, lest the albumen be accidental, and obtain a specimen passed after rising in the morning and another after a meal. If it is certainly present we must put off the candidate's acceptance for a time. His family doctor should take him in hand, see that he is clothed in woollen and gives up cold bathing out of doors, reduces the meat he eats, cuts off all stimulants, and keeps the bowels well open. He may give a hot bath before going to bed, and perhaps order with advantage the perchloride of iron. In many of these cases there is a complete recovery in 12 months, and they may then be accepted. Sesenowski[11] finds that in infants there is albuminuria in the first week in 22 per cent. It is found most often after a difficult birth, and in small children and in those where weight is lost in the first week. On the other hand, mucin exists in nearly all children, and this has misled some observers as to the frequency of albumen. Arthur Elliott[12] suggests that cyclical albuminuria may be due to a previous attack of nephritis, which has in other respects been cured, and gives details of two cases in support of this view, showing that these post nephritic cases differ in no way from others. Hauser[13] also takes the same view, and in 14 cases which he studied found a history of previous nephritis in each, following an infectious fever. He thinks that the damaged epithelium of the glomeruli fail to retain albumen under conditions of strain. Treatment by diet and prolonged rest in bed should be followed by very gradual return to muscular activity if we wish to obtain a permanent cure.

**Treatment.**—Delancy Rochester[14] argues for the use in acute nephritis with very scanty urine of vigorous cupping over the loins, venesection, hotair baths, and minute doses of pilocarpine. In subacute cases he would wash out the bowel daily with saline solution. Beverley Robinson keeps his patients wrapped up in blankets on a diluted milk or gruel diet. After a few days he allows plenty of water. He recommends high rectal injections and hot wet packs. In threatening uræmia, nitro-glycerine and sweet nitre, venesection, cupping, and saline injections are preferable to pilocarpine. Morphia may be given if the pupils are dilated, but not otherwise. Anders[15] insists on the need in chronic nephritis of a sufficiently nutritious diet. Proteid foods are necessary in moderation, red meat is as good as white, milk, fruits, green vegetables, and rice should be given freely. The effect of the diet on the albumen, on the weight of the patient, and elimination of urea, etc., should be noted. Renaut,[16] of Lyons, makes a startling statement that extract of macerated kidney has an extraordinary effect when given by the mouth. It acts as a powerful diuretic, reduces the albumen, and may even remove it altogether for a time. As Dubois proved, the antitoxin contained in it is not destroyed by the gastric juice. No harm results from its use except some skin troubles and gastric disturbance after a time, and it can be given together with other medicines. Horder[17] recommends van Noorden's dietary in acute nephritis. In extreme cases only a pint of milk daily is allowed to which a little calcium carbonate is added. Thirst is relieved by ice; water is eliminated with difficulty, and is therefore avoided; diaphoresis is encouraged. After some days milk is increased to 2½ pints, with half a pint of cream; fluids are, however, restricted as long as oliguria continues. White vegetable soups, farinaceous puddings, and white bread are then added. Later on vegetables, eggs, and meat are given, provided less than three ounces of proteid are taken daily, and when the urine is copious plenty of fluid should be taken. Diuretic drugs are useless throughout. In most cases of contracted kidney fluids should be restricted, moderate quantities of any meat should be given, but not high game or meat extracts or pepper and spices. In tubular nephritis some authorities regard salt as very injurious, increasing the œdema and albuminuria. Venesection seems to be useful against uræmia only in acute nephritis, and in exacerbations. Marsden recommends it where there is a dilated right heart. Diaphoresis is useful only when it is followed by increased diuresis, otherwise it may be actually injurious. When saline injections are given, the solution must be little more than half the strength used in surgery, so as to prevent any increase of the already high osmotic pressure. Kemp[18] lays down that cold injections into the bowel are dangerous, but hot ones (110° to 120°) produce rapid diuresis, diaphoresis, and fall of pulse tension. A double current irrigator, made by passing two catheters through a perineal pad, should be used. Three to twelve gallons may be injected at a time at a temperature of 100°, rising to 120°, the outflow running into a pail, and repeated every three hours. Extraordinary improvement has been obtained by these injections. Pressure on the outflow tube forces the fluid up the bowel.

8 Lancet. Dec. 19. 9 Ibid., Feb. 20. 10 Clin. Jour., Dec. 16. 11 Lancet, Jan. 30. 12 Med. Rec., Dec. 12. 13 Ibid., Jan. 9. 14 Ibid., Feb. 6. 15 Ibid., Oct. 10. 16 Ibid., Feb. 13. 17 Pract., Nov. 18 Med. Chron, April.

